# Tranexamic acid for haemostasis and beyond: does dose matter?

**DOI:** 10.1186/s12959-023-00540-0

**Published:** 2023-09-12

**Authors:** Tammy Lam, Robert L. Medcalf, Geoffrey C. Cloud, Paul S. Myles, Charithani B. Keragala

**Affiliations:** 1 Australian Centre for Blood Diseases, Monash AMREP Building, Monash University, Level 1 Walkway, Via The Alfred Centre, 99 Commercial Rd, Melbourne, 3004 Australia; 2 Department of Clinical Neuroscience, Central Clinical School, Monash University, Melbourne, Australia; 3 Department of Anaesthesiology and Perioperative Medicine, Alfred Hospital, Melbourne VIC, Australia; 4 Department of Anaesthesiology and Perioperative Medicine, Monash University, Melbourne VIC, Australia

**Keywords:** Tranexamic acid, Fibrinolysis, Thrombosis, Immunity, Amyloid, Pharmacology, Surgery

## Abstract

Tranexamic acid (TXA) is a widely used antifibrinolytic agent that has been used since the 1960’s to reduce blood loss in various conditions. TXA is a lysine analogue that competes for the lysine binding sites in plasminogen and tissue-type plasminogen activator impairing its interaction with the exposed lysine residues on the fibrin surface. The presence of TXA therefore, impairs the plasminogen and tPA engagement and subsequent plasmin generation on the fibrin surface, protecting fibrin clot from proteolytic degradation. However, critical lysine binding sites for plasmin(ogen) also exist on other proteins and on various cell-surface receptors allowing plasmin to exert potent effects on other targets that are unrelated to classical fibrinolysis, notably in relation to immunity and inflammation. Indeed, TXA was reported to significantly reduce post-surgical infection rates in patients after cardiac surgery unrelated to its haemostatic effects. This has provided an impetus to consider TXA in other indications beyond inhibition of fibrinolysis. While there is extensive literature on the optimal dosage of TXA to reduce bleeding rates and transfusion needs, it remains to be determined if these dosages also apply to blocking the non-canonical effects of plasmin.

## Background

### Fibrinolysis, plasminogen activation and the harnessing for therapeutic use

**Fig. 1:**
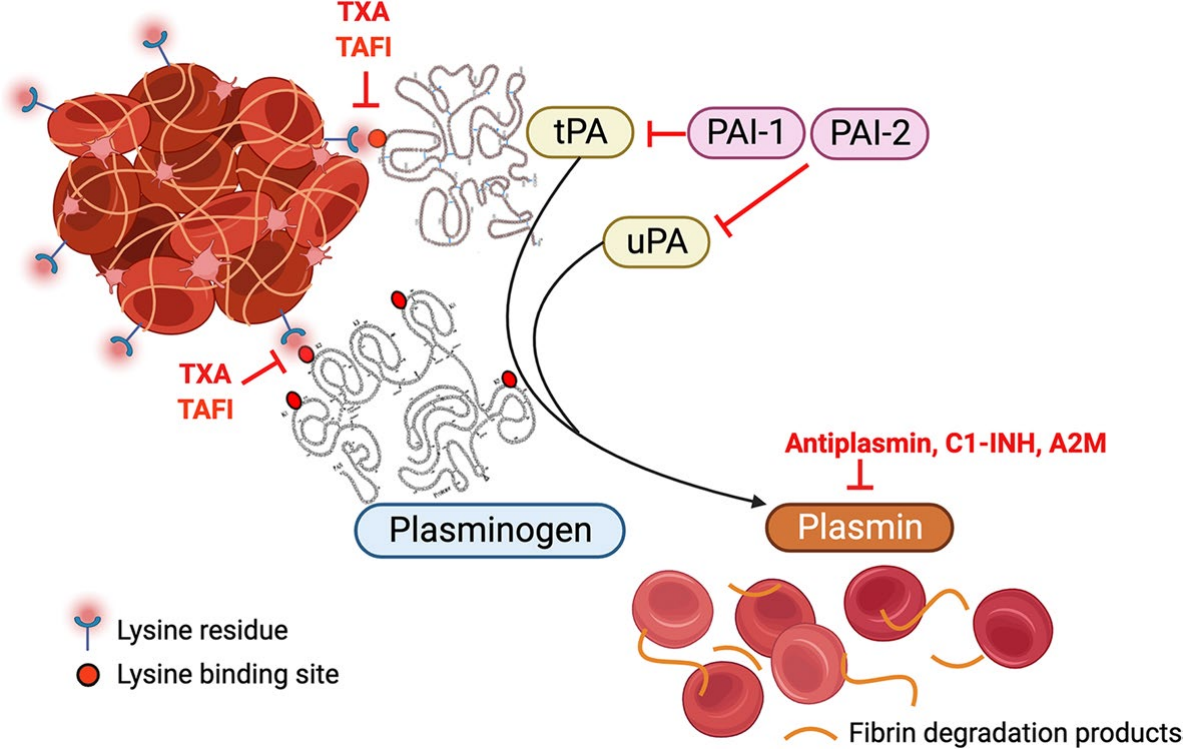
Schematic diagram of the main mechanisms of the fibrinolytic system and the target site of tranexamic acid (TXA). Lysine binding sites located within the second kringle domain of tPA, and in 4 of the 5 kringle domains of plasminogen can bind to exposed lysine residues on the fibrin surface. This interaction can be inhibited physiologically by Thrombin activatable fibrinolysis inhibitor (TAFI) or pharmacologically by competitive inhibition by the lysine analogue, TXA. The juxtaposition of tPA and plasminogen on the clot surface permits plasmin generation and subsequent fibrinolysis and the formation of fibrin degradation products. Key modulators of this process include plasminogen activator inhibitor-1, PAI-1 (and to some extent, PAI-2) that inhibit both tPA and uPA, and antiplasmin that specifically inhibits plasmin activity. Plasmin can also be inhibited by other molecules including C1-inhibitor (C1-INH) or by alpha2 macroglobulin (A2M)

Fibrinolysis is the enzymatic process underlying the removal of fibrin deposits and blood clots [1]. Fibrin comprises cross-linked fibrin monomers that provides structural integrity to blood clots. However, fibrin also harbours a means of self-destruction via the exposure of lysine residues on its surface which provides docking sites for the key fibrinolytic components plasminogen and tissue-type plasminogen activator (tPA) [2]. This then allows for the localised formation of plasmin, that executes the fibrinolytic event (Fig. 1). This fibrin-targeted feature of tPA also serves to limit systemic plasminogen activation. Plasminogen, the zymogenic precursor to active plasmin, circulates in plasma in abundance and can also be converted to plasmin by a second plasminogen activator, urokinase-type plasminogen activator (uPA) [3]. While both uPA and tPA cleave plasminogen at the same site to generate active plasmin, there are important features that separate tPA and uPA. For example, tPA is a relatively poor plasminogen activator in solution, but becomes far more efficient at activating plasminogen on the clot surface. On the other hand, uPA-mediated plasminogen activation is not at all reliant on fibrin and it could be argued that its role in the removal of fibrin deposits and blood clots is secondary to tPA [2]. The plasminogen activators are also tightly modulated to limit indiscriminate plasmin generation. Both tPA and uPA are inhibited by plasminogen activator inhibitors (PAI)-1 and PAI-2, while plasmin itself is regulated by ɑ2-antiplasmin and secondarily by C1-inhibitor (C1-INH) [4] and ɑ2-macroglobulin [5, 6].

tPA and uPA are also expressed outside the circulation and in different locations. For example, tPA is widely expressed in neurons [7] and can activate microglia [8, 9] and modulate the activity of N-methyl-D-aspartate (NMDA) receptors in the central nervous system (CNS) [10, 11]. uPA can also be found in the CNS [12, 13], but is also quite prominently expressed in extravascular compartments and is prominently expressed in the kidney [14]. This expression pattern also points to other important regulatory roles that are clearly distinct from blood clot removal. Cell-associated activities are linked to 12 known plasminogen receptors detected on most, if not all, immune cells [15]. The association of plasminogen with these cell receptors trigger downstream signalling pathways linked to immune cell recruitment, modulation of inflammation and improved wound healing [15–17].

Plasmin is also known to cleave substrates other than fibrin, such as coagulation factors (i.e. Factor V, VIII, XI, XII) and growth factors (i.e. pro-BDNF) [18, 19]. Similarly, plasmin also has the capacity to initiate non-haemostatic processes by activating the complement system, notably by cleavage of complement proteins C5, C3 and activating various metalloproteinases [20–22]. Plasmin can also target misfolded proteins for clearance including amyloid-beta (Aβ) [23, 24]. Within the CNS, tPA has been linked to various aspects of neuronal function, including the promotion of synaptic plasticity and increasing blood–brain barrier (BBB) permeability [25–28]. Some of these effects of tPA also involve plasmin generation within the CNS [29]. With such a broad array of various biological processes, plasmin and components of the fibrinolytic system appear to be an attractive target for treatment of a range of conditions.

### Targeting fibrinolysis: the critical role of lysine residues

**Fig. 2:**
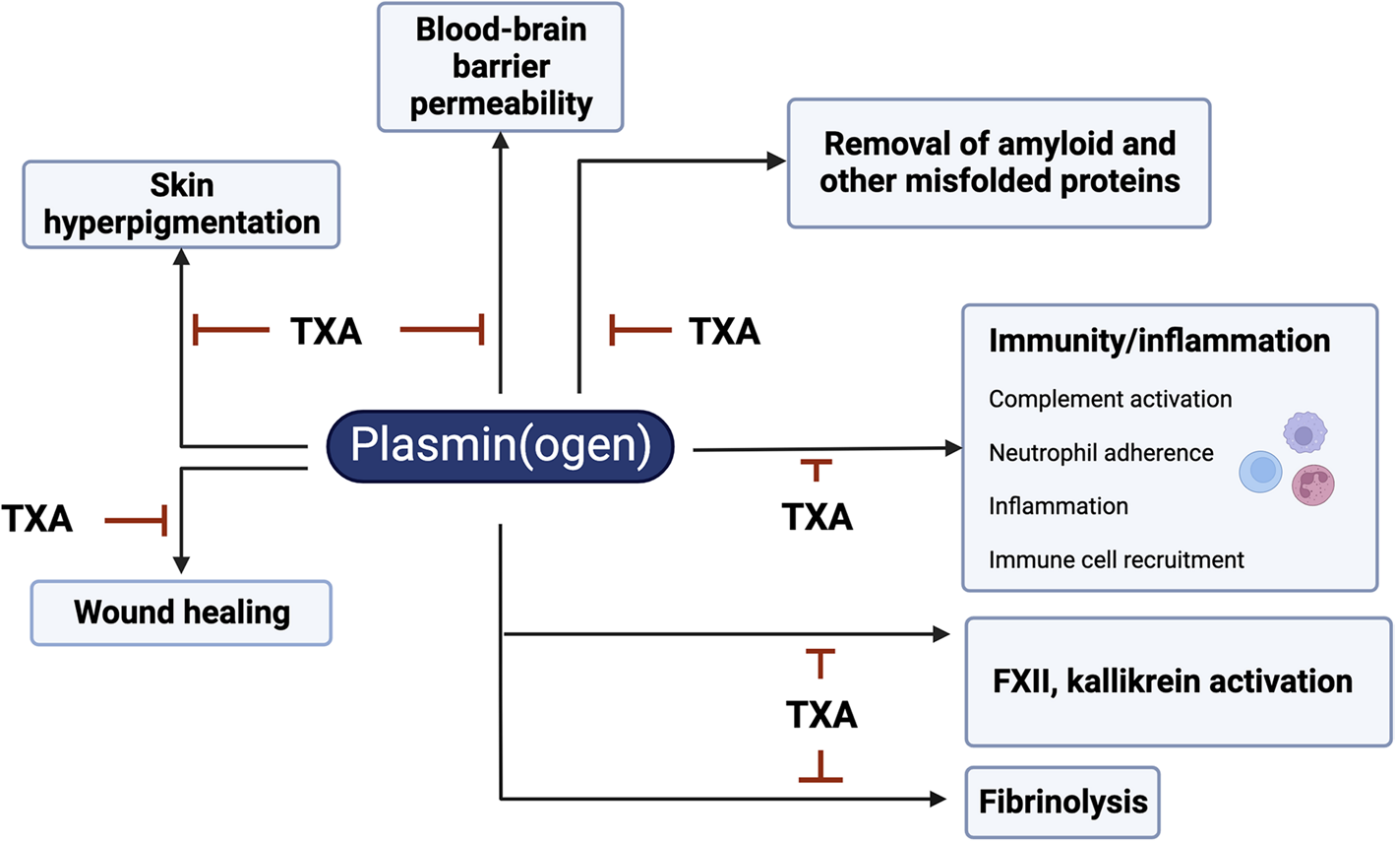
Schematic representation of the known effects of plasmin(ogen) beyond fibrinolysis and how tranexamic acid (TXA) can potentially modulate these processes

Indeed, the interaction of exposed lysine residues on the fibrin surface with lysine binding sites within both tPA and plasminogen, is key to the localized conversion of plasminogen into plasmin [2]. Up until now, the only clinical justification to modulate the plasminogen activating system has been for the purpose of either enhancing fibrin removal (i.e. thrombolysis), or to limit bleeding. Promoting fibrinolysis for myocardial infarction, pulmonary embolism or ischaemic stroke, has been attempted by the administration of high doses of exogenous tPA or uPA (or variants) to increase plasmin generation within the circulation to remove the offending blood clot. On the other side of the coin, blocking the fibrinolytic system has been achieved by interfering with the very process that facilitates the targeted binding of plasminogen and tPA to the fibrin surface, namely lysine residues. This can occur physiologically by the enzymatic removal of lysine residues from the fibrin surface by the carboxypeptidase, thrombin activatable fibrinolysis inhibitor (TAFI [30]), also known as carboxypeptidase U (CPU [31]). However, this has also been achieved pharmacologically with the use of lysine analogues that act as competitive inhibitors. These drugs were developed in Japan in the 1960s by Okamoto et al. [32, 33]. The most commonly used lysine analogues, tranexamic acid (TXA) or epsilon-aminocaproic acid (EACA), bind to the lysine binding sites in plasminogen and tPA, thereby disabling their ability to dock on the lysine residues on the fibrin surface, hence sparing fibrin from fibrinolysis. These lysine analogues (mostly TXA) have been widely used in routine haematology as effective antifibrinolytic agents in a variety of conditions associated with bleeding [34–37] (Fig. 2).

### Tranexamic acid: current usage and understanding

#### Haemostatic indications

In different countries or jurisdictions, the approved indications for TXA use varies greatly. As indicated by the Therapeutic Goods Administration (TGA) Australia, the current approved indications for TXA (intravenous) include the reduction of peri- and post-operative blood loss in cardiac surgery and total hip or knee arthroplasty [38]. Oral tablets are recommended for short-term use to treat menorrhagia, hyphaema and clotting disorders [38]. In the USA, oral TXA is only indicated for patients with haemophilia or those undergoing dental extractions [38]. Pharmacokinetic data suggests that the elimination half-life of TXA is approximately 2 h when administered either orally or intravenously [39]. Bioavailability data appears to vary amongst administration routes and other factors, ranging from 33% to about 53% [38–40].

The effectiveness of TXA in reducing surgical bleeding and transfusion requirements is well established. TXA has been shown to reduce blood transfusion requirements by 25%-60% in orthopaedic surgeries, including primary knee and hip arthroplasty [41]. These beneficial outcomes are accompanied by no evidence for an increased risk of thromboembolic complications [42]. In cardiothoracic surgery, post-operative bleeding is a common complication as a result of cardiopulmonary bypass (CPB), circulatory dilution, aggressive anticoagulation, platelet dysfunction and a systemic inflammatory response to CPB. TXA has been shown to reduce bleeding post CPB as well as transfusion requirements without any evidence of increased death or thromboembolic complications [36, 43]. A seminal trial of TXA versus placebo in coronary artery surgery was the ATACAS trial, which found that patients randomised to TXA received 46% fewer blood products than those in the placebo group. The investigators found that the use of TXA would save approximately 57 units of blood products for every 100 patients treated, which are effects similar to those reported in other meta-analyses [44]. The trial also found that TXA treatment resulted in significantly lower rates of reoperation and a general improvement in blood-sparing effects [36]. TXA was however associated with a higher risk of seizures in this patient group, possibly due to the significantly higher dose of TXA used. TXA is also used aggressively to reduce obstetric bleeding, which was in fact the original purpose and goal for the development of TXA by Dr. Okamoto. The success of TXA in reducing bleeding was demonstrated in the WOMAN trial where early administration of TXA significantly reduced post-partum haemorrhage which could have otherwise resulted in death [45].

In trauma and traumatic brain injury (TBI), it was shown that TXA safely reduces the risk of mortality from injury-related bleeding when administered within the first 3 h [46, 47]. Interestingly, when TXA was administered after 3 h, it caused an increase in bleeding and mortality. The molecular underpinnings of this has eluded the field although it is speculated to be related to the ability of TXA to paradoxically increase fibrinolytic activity via activation of uPA [48]. The recently completed PATCH-Trauma trial, where patients with severe trauma and at risk of coagulopathy were randomised to TXA by paramedics (i.e. prehospital administration) also reported reduced mortality rates, but an improved functional outcome at 6 months was not shown in the TXA group [49].

In contrast, TXA has not been shown to be effective at improving outcomes in patients with gastrointestinal bleeding in the HALT-IT trial [50, 51]. Furthermore, extended use of a high dose TXA regimen did not reduce death from gastrointestinal bleeding compared to placebo. Concerningly, there were even more venous thromboembolic events in the TXA group, with the researchers recommending that TXA should not be used for the treatment of gastrointestinal bleeding outside the context of a randomised trial [50].

Similarly, TXA was not shown to be beneficial for intracerebral haemorrhage (ICH) in improving clinical outcomes as demonstrated by the TICH-2 trial and STOP-AUST trial [51, 52]. In particular, treatment for ICH requires early administration, as haematoma expansions occur shortly after ICH, and is often a determinant of favourable outcomes [53, 54]. Although the TICH-2 trial showed fewer deaths by day 7 in the TXA treatment arm, there was no difference in mortality at 90 days between the two groups [51]. In the STOP-AUST trial, patients who suffered from acute ICH were administered TXA within a 4-5 h time window [52]. Likewise, there was no effect on the growth of ICH. Several systematic reviews however appear to suggest otherwise, indicating that TXA had successfully reduced the risk of ICH growth, although longer term mortality outcomes, rebleeding risk and length of hospital stay remained unchanged [55, 56].

Given the variable effects of TXA on bleeding and functional outcomes, this may suggest that TXA is augmenting other processes unrelated to its blood-sparing effects which should be taken into consideration.

### Haemostatic indications for TXA linked to amyloid-mediated plasmin generation

#### Cerebral amyloid angiopathy

Cerebral amyloid angiopathy (CAA) is a serious and increasingly recognised neurological condition largely diagnosed in those over the age of 50 years. CAA is caused by pathologic deposition of amyloid-beta (Aβ40) within the vessels of cortical and leptomeningeal arterioles [57, 58]. CAA results in intracranial bleeding in the form of lobar ICH, microbleeds, convexity subarachnoid haemorrhage (cSAH) and superficial siderosis [59, 60]. Several mechanisms have been proposed of how this occurs but there is evidence for BBB leakiness and inflammation due to cerebrovascular deposits of Aβ40. There are no current treatment options available for recurrent or incident stroke prevention in CAA other than generic stroke prevention lifestyle advice, lowering blood pressure and avoiding concurrent antithrombotic treatments. Existing studies of drugs that primarily target Aβ40 have not been successful [61, 62]. More recently however, TXA has been proposed as a novel means to reduce the risk of ICH in CAA. The rationale for this stems from the observation that amyloid deposits can initiate plasminogen activation in the same lysine-dependent manner as fibrin, and hence can be inhibited by TXA [63]. Indeed, the plasminogen activating system is one of the proteolytic systems involved in the clearance of amyloid [64]. Hence in CAA, amyloid deposits within cerebral vessels have the potential to generate plasmin. In this scenario, it is amyloid deposition, rather than fibrin formation that is driving plasmin generation. As plasmin has also been reported to increase BBB, TXA could provide a potential means to prevent spontaneous ICH in CAA patients [65]. While this is yet to be studied in humans, intravenous TXA was shown to be able to reduce acute hematoma expansion in a post-hoc analysis of CAA patients in the TICH-2 trial, although there was no favourable effect on clinical outcome [66].

A potential concern of using long term oral TXA as secondary stroke prevention in CAA, is that plasmin is involved in the clearance of amyloid and other misfolded proteins and that TXA may inhibit this process, causing increased Aβ40 deposition. However, a recent study evaluating the effect of long term oral TXA administration to mice predisposed to Alzheimer’s disease, did not reveal any change in Aβ40 levels in the brain [67] although more research is needed to investigate the safety and efficacy of TXA in CAA patients. Hence the stage is set to evaluate TXA as a novel means to reduce ICH in patients with CAA although the dosage and duration of TXA treatment needs to considered.

#### Non-haemostatic indications for TXA

Given plasmin(ogen)’s many non-canonical properties mentioned earlier, and the fact that many of these processes are lysine-dependent, it is not surprising that TXA may block plasmin’s ability to modulate these processes as well (Fig. 2). In some circumstances, these non-canonical effects of plasmin can be potentially deleterious [68, 69]. In the case of TBI, plasmin promotes the breakdown of laminin in the hippocampus, which subsequently triggers neuronal death [70]. At the same time, plasmin-mediated activation of the complement cascade can drive neuroinflammation and secondary injury in TBI [71]. A similar effect has also been observed in the ischemic brain [72]. As a result, there has been growing interest in the therapeutic use of TXA for purposes beyond its antifibrinolytic effects.

#### Inflammation and immunity

Plasminogen is well known to have an important role in inflammation and immunity. In the mid 1990’s, studies using plasminogen deficient mice revealed a critical role for plasminogen in immune cell (macrophage and lymphocyte) recruitment during the pro-inflammatory phase of the immune response [73]. Immune cells have a very high capacity to engage plasminogen and this is mediated by its ability to bind to plasminogen receptors on the surface of immune cells. At least 12 distinct cell surface plasminogen receptors have been described [74] and most of these contain a C-terminal lysine residue that is recognised by the lysine binding sites within the plasminogen kringle domains. Hence, TXA will block the binding of plasminogen to these receptors and interfere with intracellular signal transduction pathways initiated by direct receptor binding or via cell surface plasmin generation. This has already been demonstrated for the plasminogen receptor, Plg-RKT [17] and using Plg-RKT deficient mice in models of wound healing.

Growing evidence has now accumulated indicating the effect of TXA on inflammation and immunity in vivo. TXA has been shown to have anti-inflammatory effects attributed to plasmin inhibition [75]. More recently, Draxler and colleagues showed a reduction in postsurgical infection rates among patients undergoing cardiac surgery who were randomised to TXA compared to placebo despite all patients receiving prophylactic antibiotics [36, 76]. This effect was independent of transfusion requirements and TXA’s effect on bleeding reduction. TXA-mediated plasmin blockade was found to enhance the expression of immune-activating markers (CD83 on monocytes and CCR7 on dendritic cells) while reducing the expression of immunosuppressive markers (i.e. Programmed Cell Death Ligand-1; PDL-1) leading to improved immune competence on multiple myeloid and lymphoid populations in peripheral blood. Other studies have also shown similar results with TXA reducing levels of IL-6, IL-1β and TNF-α in patients undergoing CPB [77]. Draxler and colleagues have also shown that TXA reduces baseline IL-6, TNF-α, INF-γ and IL-10 levels in the blood of healthy subjects administered TXA [76]. A recent systematic review and meta-analysis further highlights the anti-inflammatory effects of TXA in cardiac surgery patients [78].

In orthopaedic surgery, TXA has also been shown to reduce plasma levels of CRP and IL-6 after total knee and hip arthroplasty. This may be related to both the direct and indirect effects of plasmin’s ability to activate several inflammatory pathways initiated by cell surface plasminogen receptors (above), including the complement system [79, 80].

While the capacity of TXA to influence these inflammatory processes has been associated with plasmin inhibition, TXA has also been reported to exert direct (i.e. plasmin-independent) anti-microbial effects in vitro [81, 82]. Furthermore, a more recent study reported that TXA could modulate levels of TNF and IL-1 in plasminogen deficient mice [83], again supporting the view that TXA could influence inflammation via other means.

While the above commentary relates to beneficial effects of TXA by blocking the immunosuppressive (and possibly pro-inflammatory) actions of plasmin, it also needs to be considered that elevated levels of plasmin can also exert beneficial effects, and in such a scenario, TXA treatment may counter this to the detriment of the host (see below).

Plasmin formation has also been reported to have both beneficial and detrimental effects during infection and sepsis, respectively, in infection models using plasminogen deficient mice [84]. It is unclear the mechanistic basis for the deleterious effect of plasmin in the sepsis model, but the authors of this study suggested that this might be related to a marked plasmin-mediated pro-inflammatory state, that included complement activation and subsequent STAT3 signalling, but this remains speculative. More recently, Vago et al. reported that plasmin could reduce sepsis severity by reducing inflammation and the formation of neutrophil extracellular traps in a process that was blocked by the addition of TXA [85].

#### Wound healing

As mentioned earlier, plasminogen is a key component in the wound healing process**.** Indeed, plasminogen-deficient mice display markedly delayed wound healing [86, 87] and has been reported to act as a master regulator and a potential drug candidate for the healing of radiation wounds [88]. A key question is whether the use of TXA might interfere with any lysine-dependent process associated with wound healing. Even if wound healing was delayed by TXA, this would only be evident for the duration of the TXA treatment period (usually less than 8 h) and therefore may not be clinically noticeable. On the other hand, a different scenario might occur in situations where TXA treatment is prolonged. On this note, recent case reports have provided evidence that topical TXA over 72 h slowed down wound healing during facial plastic surgery [89], however larger studies are needed to confirm this observation.

#### Melasma

For over 40 years, TXA has been used for cosmetic skin-whitening purposes [90] and more recently as a means to reduce UV-induced hyperpigmentation in melasma by inhibiting the ability of plasmin to increase melanin production in keratinocytes [91–93]. TXA seems to be able to block plasmin-mediated induction of arachidonic acid-induced pigmentation [93] while other in vitro research suggests that stimulation of autophagy by TXA as well as the suppression of VEGF-stimulated melanogenesis could also underlie the mechanism for TXA’s anti-melanogenic property [94, 95].

#### Hereditary Angioedema

Hereditary angioedema (HAE) is a potentially life-threatening condition characterised by recurrent attacks of cutaneous and submucosal angioedema as a result of mutations in the genes encoding C1-INH. Cross communication between coagulation and complement serine protease systems means that C1-INH also inhibits proteases of the coagulation, fibrinolytic and kinin pathways, being a critical physiological inhibitor of plasma kallikrein, FXIa and FXIIa [20, 96]. Edema therefore occurs as a result of excessive bradykinin production from the unregulated activation of the prekallikrein-kallikrein-HMWK-bradykinin system due to C1-INH deficiency or dysfunction [97]. TXA has been used successfully for prophylaxis in patients with HAE with the thought that it reduces the sensitivity to plasminogen cleavage, thus reducing the excess bradykinin production [98, 99] – a mechanism and clinical use completely unrelated to its haemostatic properties.

In close relation to this, TXA appears to play an important role in maintaining vascular integrity in other pathological contexts. Attenuation of the loss of endothelial cell adhesion junctions as well as maintaining the vascular-protective role of the endothelial glycocalyx are important pathways by which TXA achieves this [100].

### TXA at the cellular level

At a more cellular level, recent studies have shown that TXA decreased the release of mitochondrial DNA (mtDNA) into plasma following burn injury in mice as well as inhibiting the spontaneous release of mtDNA from human endothelial cells and neutrophils [101]. TXA was also shown to modulate various intracellular signalling pathways in these studies, although the mechanistic underpinnings remain to be determined, and also if this indeed was a consequence of inhibition of the plasminogen activating system. The same group of researchers have shown that TXA enhanced mitochondrial respiration and ATP production; important processes that could work to protect these vital cellular systems and hence the host, in the setting of marked metabolic stress and derangement seen in trauma, surgery and sepsis [102, 103].

Perhaps linked to its effects on mitochondria are the anti-aging properties of TXA published over recent years. Hiramoto et al. have demonstrated that long-term oral TXA administration suppressed age-dependent wrinkle formation in mice with hereditary skin dryness [104]. This was accompanied by increased production of hyaluronic acid, increases in epidermal cell number and reduced proteolytic degradation of the extracellular matrix. Furthermore, there was reduced mast cell proliferation, increased β-endorphin levels and increased expression of μ-opioid receptors on the surface of fibroblasts. Curiously, continuous administration of TXA significantly increased the lifespan of mice and reduced blood levels of IL-6, TNF-α, reactive oxygen species and MMP-9 as well as suppressing age-related osteoporosis and reducing apoptosis in the gonadal organs of aging mice [105, 106].

With growing evidence on the non-haemostatic effects of TXA, it is surprising that most clinical studies described in the previous section have only been directed towards the antifibrinolytic actions of TXA.

### Current trials evaluating the potential non-haemostatic benefits of TXA

The promising immune-modulatory potential of TXA is currently being investigated in the treatment of necrotizing soft-tissue infections (TRITON trial: A feasibility trial of tranexamic acid for necrotising soft-tissue infections) as well as its capacity to reduce the incidence of surgical site infection rates in patients undergoing gastrointestinal surgery (TRIGS trial: Tranexamic acid to reduce infection after gastrointestinal surgery) [107, 108]. In addition to its immune-modulating effects, plasmin also has BBB modulating capacities which can contribute to postoperative delirium. As such, a sub study of the TRIGS trial (TRIGS-D) is evaluating whether TXA might reduce neuroinflammation and subsequent postoperative delirium [108].

### Dose considerations—how much TXA is enough?

Most studies involving the use of TXA are for haemostatic indications and trials often use bleeding or mortality as the primary outcome measure. A systematic review by Picetti et al. summarized various in vitro studies of fibrinolytic inhibition by TXA [109]. It has been suggested that TXA concentrations of 10–15 mg/L were sufficient to achieve a substantial level of fibrinolysis inhibition in vitro [109]. However, it is of note that the simulated conditions in vitro are vastly different from the clinical context. Nevertheless, these in vitro studies may still provide some guidance and insight into dosing regimens for fibrinolytic inhibition.

In patients, dosage of TXA appears to vary largely between procedures and/or clinical conditions. In particular, administration of TXA to patients undergoing cardiac surgery generally uses uniquely high doses of the drug at up to 100 mg/kg [110]. The ATACAS trial demonstrated reduced bleeding-related complications in cardiac surgery using a dose of 50–100 mg/kg, and post hoc analyses demonstrated likely immune-enhancing properties at this higher dose [110]. Although this provides additional benefit in reducing blood loss and infections, some observational studies have reported that higher doses can also increase the risk of postoperative seizures [36, 110]. The recent OPTIMAL randomised clinical trial compared the efficacy and adverse events of high-dose (30 mg/kg bolus, a 16 mg/kg/h maintenance dose, and a 2 mg/kg prime) versus low-dose tranexamic acid (10 mg/kg bolus, a 2 mg/kg/h maintenance dose, and a 1 mg/kg prime) in patients undergoing cardiac surgery with CPB [43]. This study found that patients receiving the high-dose TXA infusion had a statistically significant reduction in the proportion of patients requiring a blood transfusion [43]. Furthermore, the study results met criteria for noninferiority with respect to composite primary safety endpoints of 30-day mortality, seizure, kidney dysfunction, and thrombotic events [43].

In the context of trauma, patients who received repeated dosing of TXA (3 g in total) had a significantly lower mortality rate compared to placebo (STAAMP trial) [111]. Patients in the CRASH-2 and 3 trials who received a total of 2 g of TXA within 3 h from injury had a significantly reduced risk of bleeding-related deaths [46, 47].

However, as seen in the previously described studies, the potential for TXA to block the non-haemostatic effects of plasmin have not been accounted for and may have provided further benefit but had gone unnoticed. However, there is still a small pool of completed and ongoing studies that utilize TXA for non-haemostatic indications – what remains largely unknown is whether the dosages used are appropriate to block these non-haemostatic pathways of plasmin(ogen).

In patients with melasma, a recent network meta-analysis has suggested that the optimal treatment would be a total daily oral dosage of 500–750 mg for 12 weeks. In this analysis, it was also suggested that treatment duration could influence treatment outcome. However, it is not known if treatment durations beyond 12 weeks would provide extra benefit [112].

In the ongoing trial for necrotizing fasciitis (TRITON), intravenous TXA is administered for a total of 2 g daily for 4 days [107]. The primary outcome of this trial would be a reduction of at least 25% of the initial extent of infection.

In orthopaedic surgery, TXA-associated immune modulation was described in patients undergoing total knee arthroplasty (TKA) [113]. In this trial, only patients receiving a total of more than 6 g of TXA displayed anti-inflammatory effects until 72 h post-treatment [113]. In another trial, patients who underwent total hip arthroplasty and received a total of 3 g of TXA revealed a diminished inflammatory response at 3 days post-surgery [114]. Notably, a regime of four oral doses of TXA (2 g initial + 1 g subsequently) was found to promote both optimal blood loss reduction and a diminished inflammatory response post-operatively following TKA [115]. In another study, patients receiving a high initial dose of TXA (60 mg/kg) with 5 subsequent doses of 1 g over 24 h demonstrated a reduction in inflammatory markers as well as D-dimers [116]. It appears that repeated dosing regimens have been commonly used for patients undergoing orthopaedic surgeries.

Conversely, in trauma patients with severe injury it was found that the reduction in HLA-DR expression on monocytes was not associated with TXA administration up until 72 h post-treatment (2 g and 4 g intravenous bolus) (TAMPITI trial) [117]. This study ultimately found that TXA at this dose had minimal immune modulatory effects with respect to leukocyte and circulating cytokine levels. However, it is important to note that trauma causes a sharp decline in HLA-DR expression even prior to treatment, which can potentially mask the effects of TXA, if any.

This is in contrast to patients who underwent cardiac surgery where TXA administration was found to be immune-activating instead [76]. In the same study, it was also demonstrated in healthy volunteers that a 1 g TXA dose was able to promote immune activating effects at 24 h after administration [76]. These varied findings confirm TXA’s fibrin-independent actions.

Collectively, there appears to be a variable effect of TXA when it comes to its non-haemostatic effects in different clinical settings. We postulate that perhaps the dose and timing of TXA plays an important role in preventing certain lysine-dependent interactions over others. Whether higher doses of TXA are required to observe the immune-modulatory properties in addition to the antifibrinolytic effects warrants further investigation. A summary of the doses of TXA used in haemostatic and non-haemostatic indications is provided in Table 1.

**Table 1:** Dose of TXA used for haemostatic and non-haemostatic indications

	**Clinical Indication**	**Dose**	**References**	**Notes**
**Haemostatic indications**	Orthopaedic surgery	high dose IV TXA: ≥ 20 mg/kg or > 1 g and high dose topical TXA: > 1.5 g	[118, 119]	
	Cardiac surgery	50 mg/kg bolus	[36]	
	Trauma	IV 1 g bolus (± pre-hospital) then 1 g infusion over 8 h	[34, 49, 120]	
	Obstetric bleeding	IV 1 g bolus (± second bolus if ongoing/recurrent bleeding)	[37]	
	Dental bleeding in patients with a bleeding disorder	TXA mouthwash (50 mg/mL): 10 mL 4 times/day IV: 0.5–1 g 2–3 times/day Oral: 1–1.5 g 2–3 times/day	[121, 122]	
	Hereditary haemorrhagic telangiectasia	3 g in 24 h	[123]	
	Gastrointestinal bleeding	1 g over 1 h then 3 g over 24 h	[124]	Negative study
	Intracerebral haemorrhage	1 g over 10 min then 1 g over 8 h	[52, 55, 125, 126]	Negative study
	Malignant thrombocytopenia	1 g IV 8 hourly or 1.5 g orally 8 hourly	[127]	Negative study
**Haemostatic indications under investigation**	Reduce recurrent cerebral bleeding in chronic amyloid angiopathy	20 mg/mL for 6 months	[67]	Evaluated in mice overexpressing the mutant human amyloid precursor protein
**Non-haemostatic indications**	Hereditary angioedema	2–4 g in 24 h	[128]	Can be used therapeutically and as prophylaxis
	Melasma	500–750 mg in 24 h	[112]	
**Non-haemostatic indications under Investigation**	Reduce infection after gastrointestinal surgery	15 mg/kg bolus before surgical incision, then infusion at 5 mg/kg/h until the end of surgery		TRIGS Clinical Trial https://www.trigs.org.au/
	Reduce delirium following gastrointestinal surgery	15 mg/kg bolus before surgical incision, then infusion at 5 mg/kg/h until the end of surgery		TRIGS-D Clinical Trial https://www.trigs.org.au/trigs_d
	Reduce severity of necrotising deep tissue infection	2 g daily for 4 days		TRITON Clinical Trial https://www.anzctr.org.au/Trial/Registration/TrialReview.aspx?id=382739&isReview=true

*IV* intravenous

This concept will be an important consideration in the interpretation of results arising from the TRIGS trial, once it is completed. What makes this study unique is that in contrast to other TXA studies, the primary outcome of the TRIGS trial is surgical site infection with transfusion requirements being a secondary outcome measure. The dose of TXA used in the TRIGS trial is a bolus of 15 mg/kg prior to surgical incision followed by an infusion of 5 mg/kg/h until the end of surgery. These doses are lower than the doses used in cardiac surgery and the ATACAS study (50 mg/kg) where changes to immune profiling and surgical site infections were seen. It will be interesting to see whether the lower dose in TRIGS translates to any changes in the non-haemostatic outcomes being measured.

## Conclusion

The current knowledge of treatment regimens and dosage suggestions for TXA are based on achieving optimal antifibrinolytic effects. What remains unknown is the immune- and other impacts of TXA at the indicated dosages commonly used to exert its haemostatic effects. The currently available literature is scarce and often involves small, single-centre clinical trials. In future, it is essential for the non-haemostatic effects of TXA to be recognised and considered alongside its antifibrinolytic properties in prospective clinical studies. Similarly, it is important to determine the extent of whether the beneficial effect of TXA at reducing mortality in trauma and in other conditions may not only be due to its effect at reducing bleeding, but actually a consequence of blocking one of the many non-haemostatic effects of plasmin.

This knowledge may ultimately help determine the most appropriate dose and timing of TXA to achieve optimal haemostatic and non-haemostatic outcomes in different clinical settings.

## Data Availability

NA.
